# Supplementary material for: HO-1089 and HO-1197, Novel Herbal Formulas, Have Antitumor Effects via Suppression of PLK1 (Polo-like Kinase 1) Expression in Hepatocellular Carcinoma
Source: Cancers (Basel). 2023 Jan 30;15(3):851. doi: 10.3390/cancers15030851 (PMC9913440; doi:10.3390/cancers15030851)
Supplement: Supplementary file 1 [file cancers-15-00851-s001.zip › cancers-2103009-supplementary.pdf]

Supplementary Figure

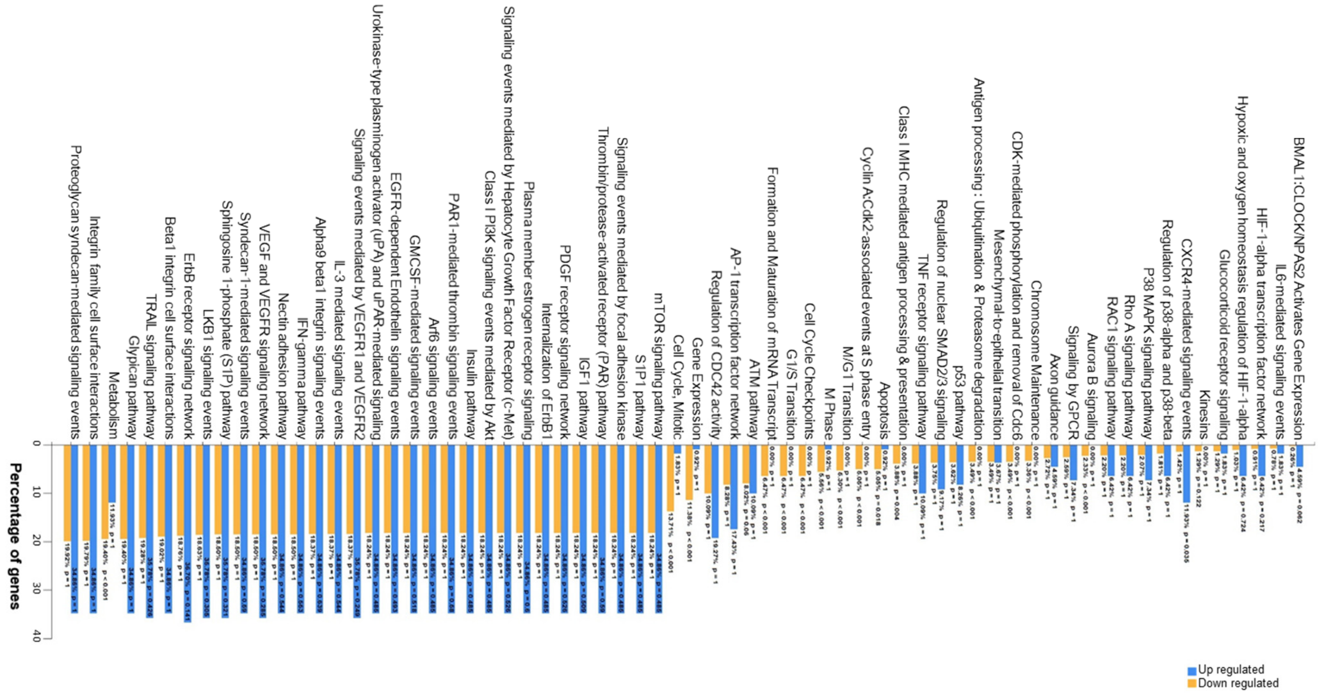

Supplementary Figure S1. Biological pathways of HO-1089 in Huh7

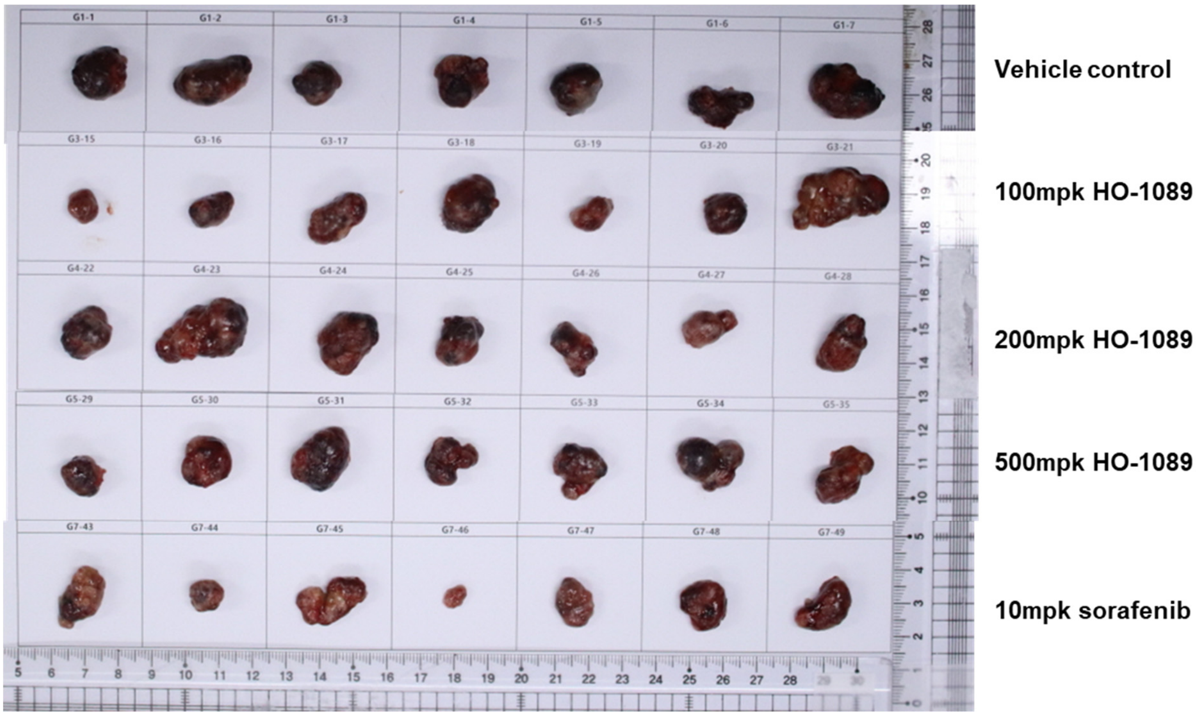

Supplementary Figure S2. Xenograft tumor after treatment of HO-1089.

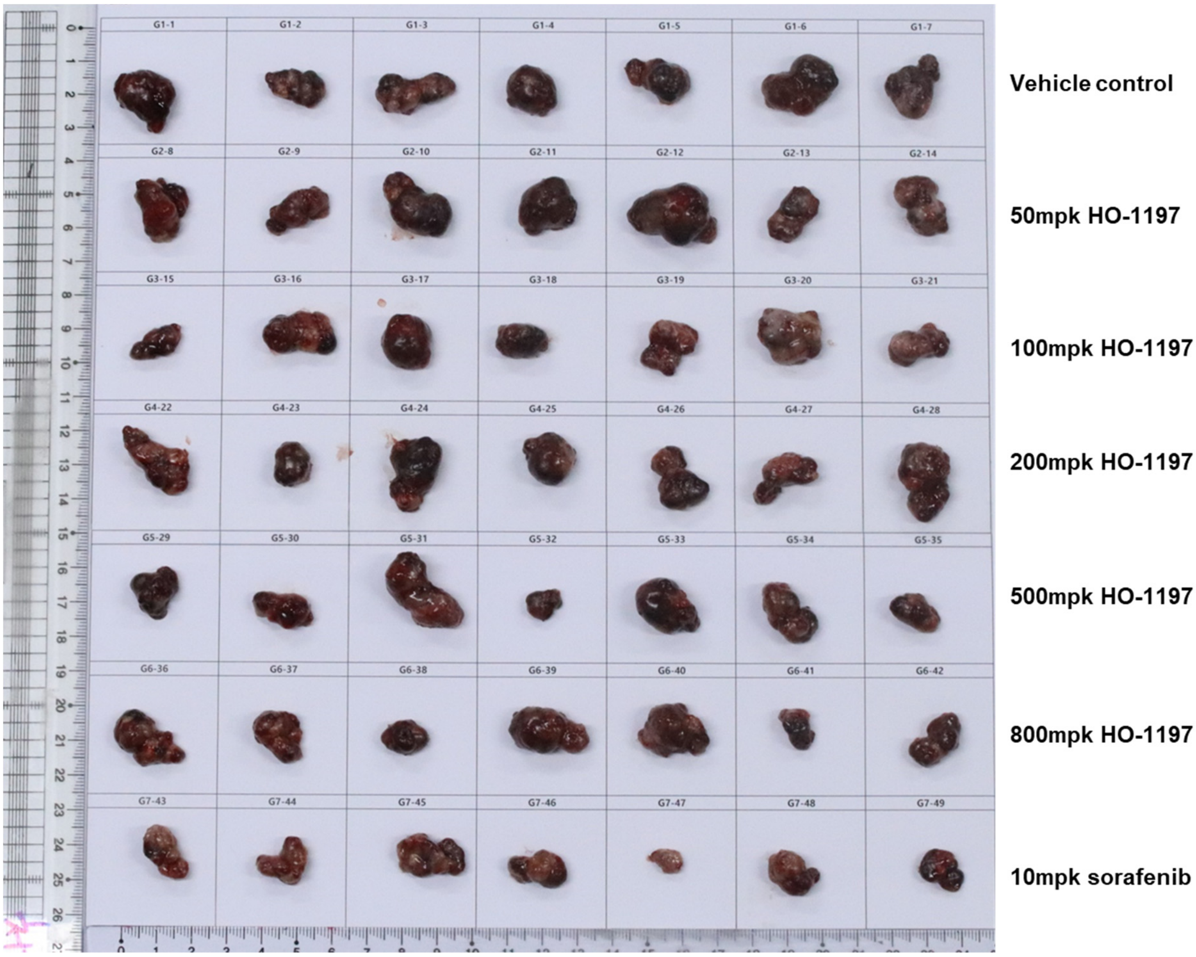

Supplementary Figure S3. Xenograft tumor after treatment of HO-1197.

Supplementary Table

	IC50 (g/ml)
H1 (HO-1089)	2.757
H2	2.95
H3	3.863
H4	6.373
H5	3.772
H6	6.854
H7	2.724
H8	3.468
H9	6.386
H10	4.044
H11 (HO-1097)	2.395
H12	3.715
H13	4.901

Supplementary Table S1. IC50 of HO-1089 (H1) and their fractions in Huh7.
